# Decreased circulating follicular regulatory T cells in patients with dilated cardiomyopathy

**DOI:** 10.1590/1414-431X2021e11232

**Published:** 2021-10-18

**Authors:** Xixi Liu, Wencai Zhang, Zhanying Han

**Affiliations:** 1 Department of Cardiology, The First Affiliated Hospital of Zhengzhou University, Zhengzhou, Henan, China; 2 Academy of Medical Sciences of Zhengzhou University, Zhengzhou, Henan, China

**Keywords:** Dilated cardiomyopathy, Follicular regulatory T cells, Immune response, Heart failure

## Abstract

Follicular regulatory T cells (Tfr) have critical functions in inflammatory and autoimmune disorders. The main purpose of the current work was to assess Tfr cell frequency in patients with dilated cardiomyopathy (DCM). Flow cytometry showed that, compared with normal controls, DCM cases showed markedly reduced Tfr cell rates and Tfr/Tfh ratios, but significantly increased follicular helper T cell (Tfh) rates. Correlation analysis showed that the Tfr rate in DCM patients was positively correlated with left ventricular ejection fraction (LVEF), and negatively correlated with N-terminal brain natriuretic peptide (NT-proBNP) levels. Lower Foxp3 and higher Bcl-6, ICOS, and PD-1 mRNA expression levels were found in patients with DCM. In addition, plasma interleukin (IL)-6, tumor necrosis factor (TNF)-α, and IL-21 levels were significantly increased in DCM cases. Moreover, IgG and IgG3 levels were also elevated in individuals with DCM. Correlation analysis showed that the Tfr rate in DCM patients was negatively correlated with IgG and IgG3, while the Tfh rate was positively correlated with IgG and IgG3. Changes in circulating Tfr levels may have a critical immunomodulatory function in DCM and may become a new therapeutic target for DCM.

## Introduction

Dilated cardiomyopathy (DCM) is a heterogeneous cardiomyopathy with unknown etiology and progressive characteristics, mainly featuring an enlarged chamber and impaired systolic function in at least one ventricle (1). Its prognosis is extremely poor, with 5- to 10-year survival rates of only 30-40% (2). Recently, multiple reports revealed that immunological factors have critical functions in patients with DCM, and have demonstrated that abnormal autoimmune reactions cause impaired activation of humoral and cellular immunity, thereby participating in the development of DCM (3,4). Activation markers, such as CD69, CD25, HLA-DR, and CD40 ligand, on the surface of T lymphocytes are significantly increased in DCM patients (5). Moreover, it was found that T lymphocytes are involved in the pathological process of DCM by direct cytotoxic effects, increasing the function of other immune cells, or assisting B cells to increase the production of pathogenic antibodies (6). Cytokines, including interleukin (IL)-6 and tumor necrosis factor (TNF)-α, are significantly elevated in DCM and show negative correlations with heart function (7). Meanwhile, anti-myocardial autoantibodies, anti-mitochondrial antibodies, and anti-β1Rs antibodies all have essential functions in DCM development, diagnosis, and treatment (8,9).

Studies have shown that changes in follicular regulatory T cells (Tfr) rate and Tfr-to-follicular helper T cells (Tfh) ratio in autoimmune pathologies have essential roles in immune tolerance and normal body immune function (10). Tfh cells, derived from conventional helper T (CD4^+^T) cells, provide essential assistance in the germinal center response and activation, proliferation, differentiation, and antibody production of B cells. High PD-1, CXCR5, ICOS, and IL-21 levels on the surface of Tfh cells enable them to be distinguished from other CD4^+^T cell subsets (10). Recently, several studies have identified a special group of Tregs (regulatory T cells) in the germinal center, namely Tfr cells. Although they have opposite effects with Tfh in regulating humoral immunity, they simultaneously express CXCR5, Foxp3, PD-1, and other markers on the surface, have the same characterization as Tfh and Tregs, and specifically inhibit Tfh and B cells for controlling germinal center reaction (11,12). Increasing evidence suggests that Tfr frequency and Tfr/Tfh ratio changes are closely associated with autoimmune diseases. For instance, Wang et al. (13) found reduced Tfr rate and Tfr/Tfh ratio in peripheral blood specimens from rheumatoid arthritis cases, and an imbalance between Tfh and Tfr cells would lead to abnormal production of autoantibodies and impaired immune tolerance. Xu et al. (14) found that Tfr rate and Tfr/Tfh ratio in peripheral blood samples from systemic lupus erythematosus cases are decreased and inversely proportional to disease severity, IL-21 levels, and anti-dsDNA antibody levels. Similar phenomena have also occurred in patients with myasthenia gravis, multiple sclerosis, primary biliary cholangitis, and systemic lupus erythematosus (10).

At present, several studies have confirmed that DCM occurrence and development have significant associations with autoimmune disorders (3). However, the rates of Tfr cell subsets and Tfr/Tfh ratio, as well as their correlation with DCM, have not been investigated. In this study, we analyzed Tfr rates in peripheral blood specimens from DCM cases by flow-cytometry, and the change of Tfr cell frequency in DCM was further discussed.

## Material and Methods

### Patients

In this study, the subjects were divided into two groups by the case-control method. Totally, 30 DCM cases (22 men and 8 women; 50±3.2 years old) newly hospitalized at the First Affiliated Hospital of Zhengzhou University (China) from May 2019 to January 2020, and 30 age- and sex-matched healthy donors (16 males and 14 females; 49.6±2.2 years old) were enrolled. DCM diagnosis followed the World Health Organization guidelines (15). DCM criteria were left ventricular end-diastolic diameter (LVEDD) ≥60 mm and left ventricular ejection fraction (LVEF) ≤40%, obtained via echocardiography, excluding ischemic cardiomyopathy, hypertensive cardiomyopathy, valvular cardiomyopathy, and congenital heart disease. Exclusion criteria were: serious inflammatory diseases, systemic autoimmune diseases, malignant tumors, liver and/or kidney dysfunctions, pregnancy, endocrine disease, and previous immunosuppressive therapy. The trial followed the principles outlined in the Declaration of Helsinki and had approval from the Ethics Committee of the First Affiliated Hospital of Zhengzhou University (Permit Number: 2019-KY-005). Written informed consent was provided by both DCM patients and healthy volunteers prior to enrollment.

### Sample collection and isolation of PBMCs

Blood specimens from DCM cases upon admission were collected in 5-mL lithium heparin blood collection tubes and centrifuged at 500 *g* at room temperature for 10 min. The resulting plasma was stored at -20°C for further measurements. Peripheral blood mononuclear cells (PBMCs) were obtained by Ficoll-Paque Plus (GE Healthcare 17-1440-03, USA) density gradient centrifugation at 500 *g* at room temperature for 10 min. Before use for flow cytometry analysis, the cells were washed twice.

### Flow cytometry

PBMCs underwent incubation with anti-human CD4-FITC (Clone A161A1), CD25-PE/CY7 (Clone BC96), CD127-APC (Clone A019D5), and CXCR5-PE (Clone J252D4) (all from BioLegend, USA), at 4°C in the dark for 30 min; the corresponding isotype controls were utilized for specificity verification. After surface staining, the FACSARIAIII flow cytometer (BD Bioscience, USA) was used for detection of cells. Then, flow cytometry data were analyzed by FlowJo 7.6.1 (Treestar Inc., USA) (Supplementary Figure S1).

### Real-time PCR

Total RNA extraction from the isolated target cells utilized TRIzol reagent (Takara, Japan), and reverse transcription was carried out with a PrimeScript^TM^ RT kit (Takara) according to manufacturer instructions. TB Green Premix EX Taq^TM^ II (Takara) was used to quantify the expression of target genes such as Foxp3 on a QuantStudio^TM^5 Real-Time PCR System (Applied Biosystems, USA). Table 1 shows primers used, designed by Primer Premier 5 (premierbiosoft.com). Triplicate assays were amplified in 45 cycles. GAPDH was utilized for normalization using 2^-ΔΔCT^ methods.

**Table 1:** Primer sequences used for RT-PCR.

Gene	Primer Sequences (5′-3′)
ICOS	
Forward	ACAACTTGGACCATTCTCATGC
Reverse	TGCACATCCTATGGGTAACCA
Bcl-6	
Forward	GGAGTCGAGACATCTTGACTGA
Reverse	ATGAGGACCGTTTTATGGGCT
Foxp3	
Forward	GTGGCCCGGATGTGAGAAG
Reverse	GGAGCCCTTGTCGGATGATG
PD-1	
Forward	ACGAGGGACAATAGGAGCCA
Reverse	GGCATACTCCGTCTGCTCAG
GAPDH	
Forward	CAGGAGGCATTGCTGATGAT
Reverse	GAAGGCTGGGGCTCATTT

### Measurement of plasma IL-6, TNF-α, IL-21, and IL-10

IL-6, TNF-α, IL-21, and IL-10 levels in plasma specimens were examined with a specific ELISA kit (Boster, China), as directed by the manufacturer. Two duplicate wells were set for each sample to reduce test errors.

### Measurement of IgG and IgG3 expression levels in plasma

Specific ELISA kits (Elabscience, China) were used to measure the expression levels of IgG and IgG3 in plasma specimens from the enrolled subjects as directed by the manufacturer. The sensitivities of the above kits were 0.94 ng/mL and 18.75 ng/mL, respectively. Each measurement was performed in duplicate.

### Statistical analysis

Data are reported as means±SE, and were compared by 2-tailed unpaired Student's *t*-test. Ratios were compared by Pearson's chi-squared test. One-way ANOVA was used for multi-group comparisons. The Pearson correlation method was utilized for assessing correlations between pairs of variables. SPSS 21.0 (IBM, USA) was employed for data analysis, with P<0.05 indicating statistical significance.

## Results

### Baseline patient features

Table 2 summarizes the patient baseline features. No significant differences were observed in age, gender, hypertension, and diabetes between the two groups. However, compared with healthy controls, DCM cases had reduced LVEF and higher LVEDD and NT-Pro-BNP levels. In the DCM group, diuretics, cardiotonic drugs, β-receptor blockers, angiotensin receptor enkephalinase inhibitors (ARNI), angiotensin-converting enzyme inhibitors (ACEI), and/or angiotensin receptor blockers (ARB) were utilized at higher frequencies compared with the healthy control group.

**Table 2:** Baseline characteristics of all enrolled subjects.

Baseline characheristics	DCM group (n=30)	Control group (n=30)	P-value
Gender (male/female)	22/8	16/14	0.108
Age (years)	50.0±3.2	49.6±2.2	0.905
NYHA (II/III/IV)	9/8/13	-	-
LVEF (%)	26.4±1.5	62.9±0.7	<0.001
LVEDD (cm)	70.2±1.6	45.9±1.1	<0.001
Hypertension, n (%)	9 (30)	13 (43.3)	0.284
Diabetes, n (%)	6 (20)	3 (10)	0.278
NT-proBNP (pg/mL)	3439.4±576.6	250.9±50.5	<0.001
Medication, n (%)			
ARNI/ACEI/ARBs	21 (70)	8 (26.7)	0.001
β-Blocker	21 (70)	5 (16.7)	<0.001
Digitalis	24 (80)	0 (0)	<0.001
Diuretics	30 (100)	0 (0)	<0.001

The data are reported as means±SE or numbers (percentages) (*t*-test or chi-squared test). DCM: dilated cardiomyopathy; NYHA: New York Heart Association; LVEF: left ventricular ejection fraction; LVEDD: left ventricular end-diastolic dimension; NT-proBNP: N-terminal pro-B-type natriuretic peptide; ARNI: angiotensin receptor enkephalinase inhibitor; ACEI: angiotensin converting enzyme inhibitor; ARB: angiotensin receptor blocker.

### CD4^+^CD25^+^CXCR5^+^CD127^-/lo^ Tfr and CD4^+^CXCR5^+^CD127^+^ Tfh cell rates in DCM patients

**Figure 1:**
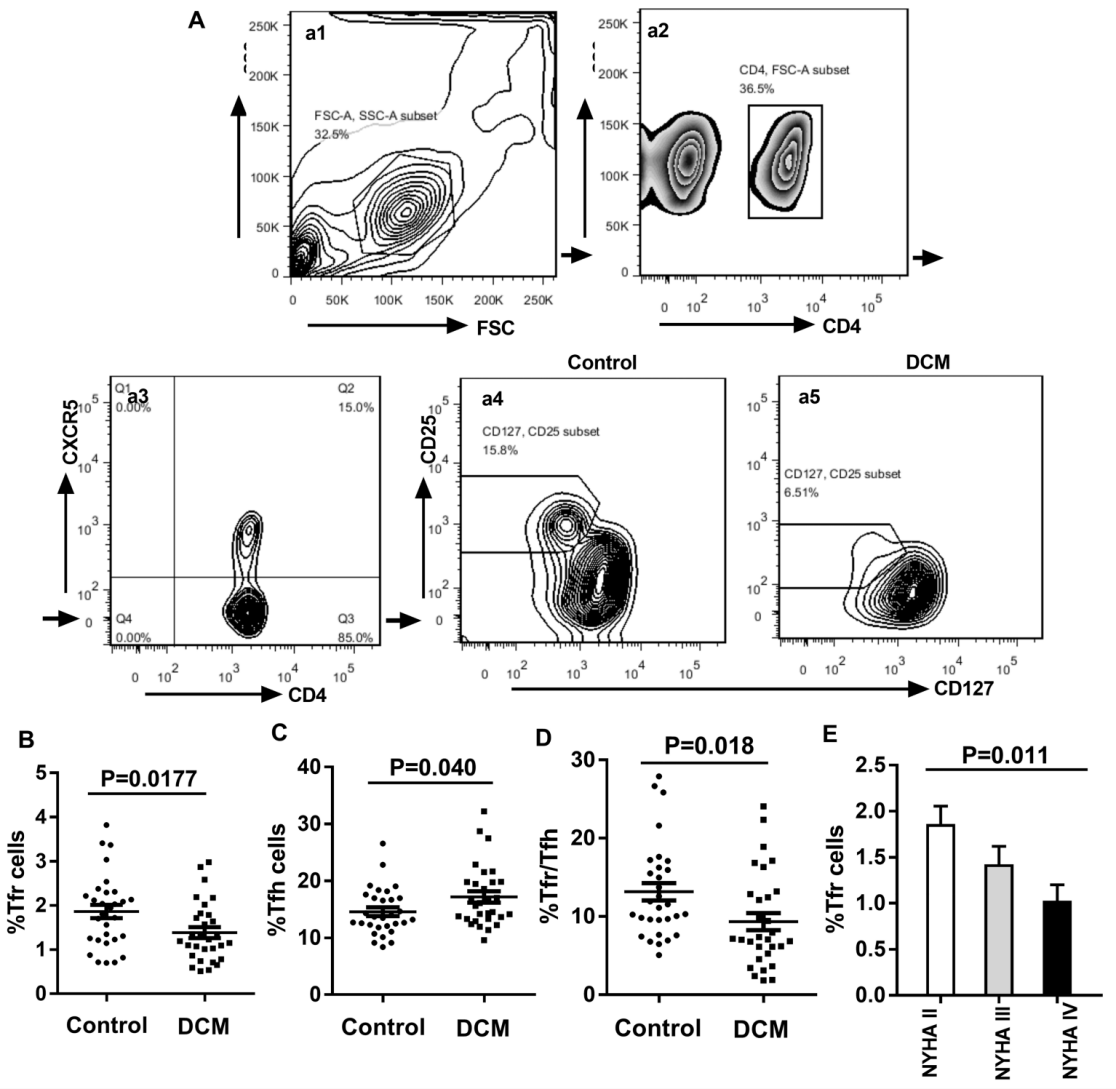
Decreased frequency of circulating CD4^+^CD25^+^CXCR5^+^CD127^-/lo^ Tfr in peripheral blood mononuclear cells (PBMCs) from patients with dilated cardiomyopathy (DCM). **A**, Representative FACS images of the frequency of CD4+CD25+CXCR5+CD127-/lo Tfr and CD4+CXCR5+CD127+ Tfh from a single patient in each group: (**a1**) the lymphocytes were first gated; (**a2**) the CD4^+^ T cells were gated; (**a3**) the expression of CXCR5^+^ was analyzed in the gated CD4^+^ T cells, and (**a4**, **a5**) the expression of CD25^+^CD127^-/lo^ was analyzed in the gated CD4^+^CXCR5^+^ T cells. **B**-**D,** the percentages of CD4^+^CD25^+^CXCR5^+^CD127^-/lo^ Tfr in CD4^+^ T cells and CD4^+^CXCR5^+^CD127^+^ Tfh in CD4^+^ T cells, and the ratio of Tfr/Tfh in DCM patients. Data are reported as means±SE. All were compared with healthy controls by the *t*-test. **E**, Tfr rate decreased with the increase of heart failure grade (ANOVA). NYHA: New York Heart Association (grades II/III/IV).

Based on the levels of cell surface biomarkers, the cells were divided into the CD4^+^CD25^+^CXCR5^+^CD127^-/lo^ Tfr and CD4^+^CXCR5^+^CD127^+^ Tfh groups; the gating strategies are shown in Figure 1A. According to the results obtained, the DCM group had a decreased proportion of CD4^+^CD25^+^CXCR5^+^CD127^-/lo^ Tfr cells (P=0.0177, Figure 1B) and increased rate of CD4^+^CXCR5^+^CD127^+^ Tfh cells (P=0.040, Figure 1C) compared with healthy controls. Tfr/Tfh ratios were also decreased significantly in DCM cases versus control patients (P=0.018, Figure 1D). In addition, we compared the frequency of Tfr cells in peripheral blood of three types of heart failure patients based on NYHA classification, and our results showed that the Tfr rate decreased with an increase of heart failure grade (P=0.011, Figure 1E). These results are consistent with previous studies (16).

### Associations of cardiac function indexes with Tfr and Tfh cell rates in DCM patients

**Figure 2:**
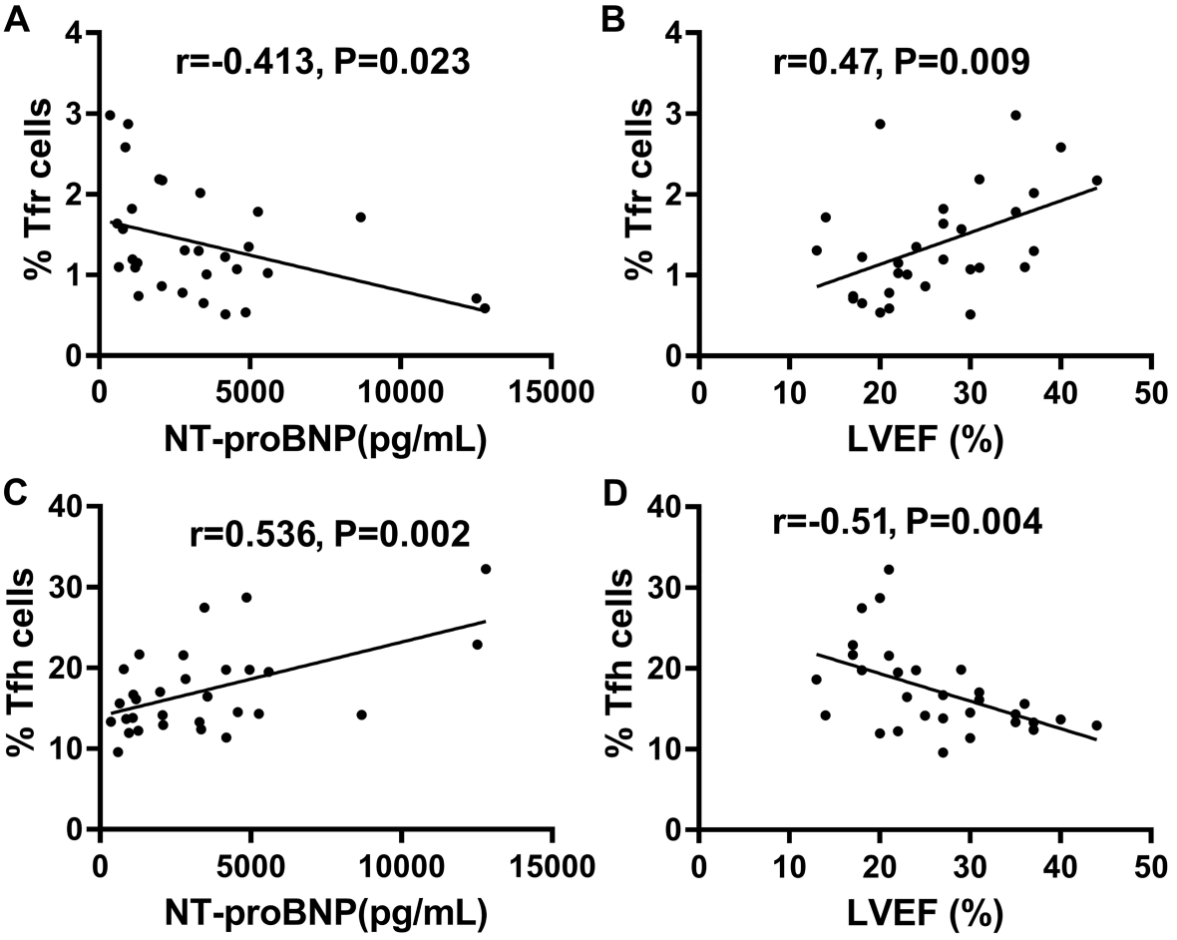
Pearson correlation of follicular regulatory T cells (Tfr) frequency, follicular helper T cells (Tfh) frequency, and indices of cardiac function in dilated cardiomyopathy (DCM) patients. The frequency of CD4^+^CD25^+^CXCR5^+^CD127^-/lo^ Tfr was negatively correlated with NT-proBNP (**A**) and positively correlated with left ventricular ejection fraction (LVEF) (**B**) in DCM patients. The frequencies of CD4^+^CXCR5^+^CD127^+^ Tfh were positively correlated with NT-proBNP (**C**) and negatively correlated with LVEF (**D**) in DCM patients.

Next, we analyzed the association of CD4^+^CD25^+^CXCR5^+^CD127^-/lo^ Tfr and CD4^+^CXCR5^+^CD127^+^ Tfh cell rates with heart function in DCM cases. As shown in Figure 2, Tfr cell rate in DCM patients had a positive correlation with LVEF (r=0.47, P=0.009; Figure 2B) and a negative correlation with NT-proBNP (r=-0.413, P=0.023; Figure 2A). Meanwhile, CD4^+^CXCR5^+^CD127^+^ Tfh cell rate had negative and positive correlations with LVEF (r=-0.51, P=0.004; Figure 2D) and NT-proBNP (r=0.536, P=0.002; Figure 2C), respectively.

### Decreased Foxp3 mRNA expression levels and elevated mRNA Bcl-6, ICOS, and PD-1 levels in DCM cases

**Figure 3:**
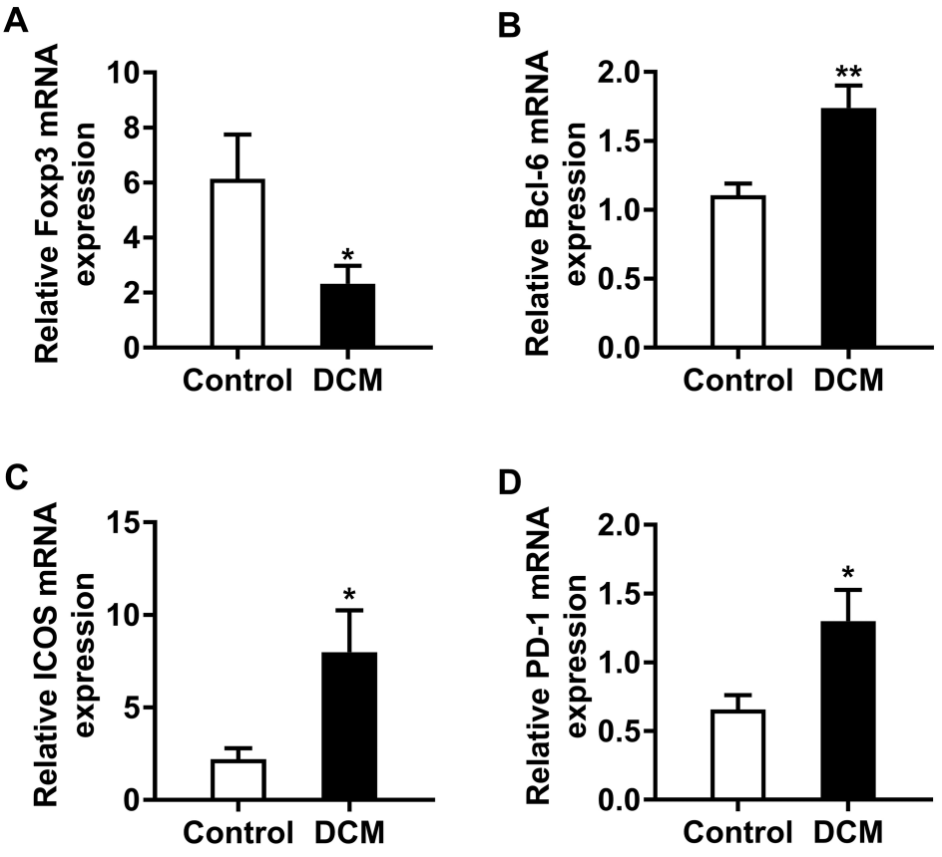
The mRNA expression levels of important molecules in peripheral blood mononuclear cells from dilated cardiomyopathy (DCM) patients and controls: Foxp3 (**A**), Bcl-6 (**B**), ICOS (**C**), and PD-1 (**D**). Data are reported as means±SE (n=12). *P<0.05, **P<0.01 compared to the Control group (*t*-test).

Our results showed that Bcl-6, ICOS, and PD-1 mRNA levels in PBMCs isolated from DCM cases were markedly elevated compared with control values (Figure 3B-D), while Foxp3 mRNA levels were significantly decreased (Figure 3A).

### Levels of cytokines in DCM and control patients

**Figure 4:**
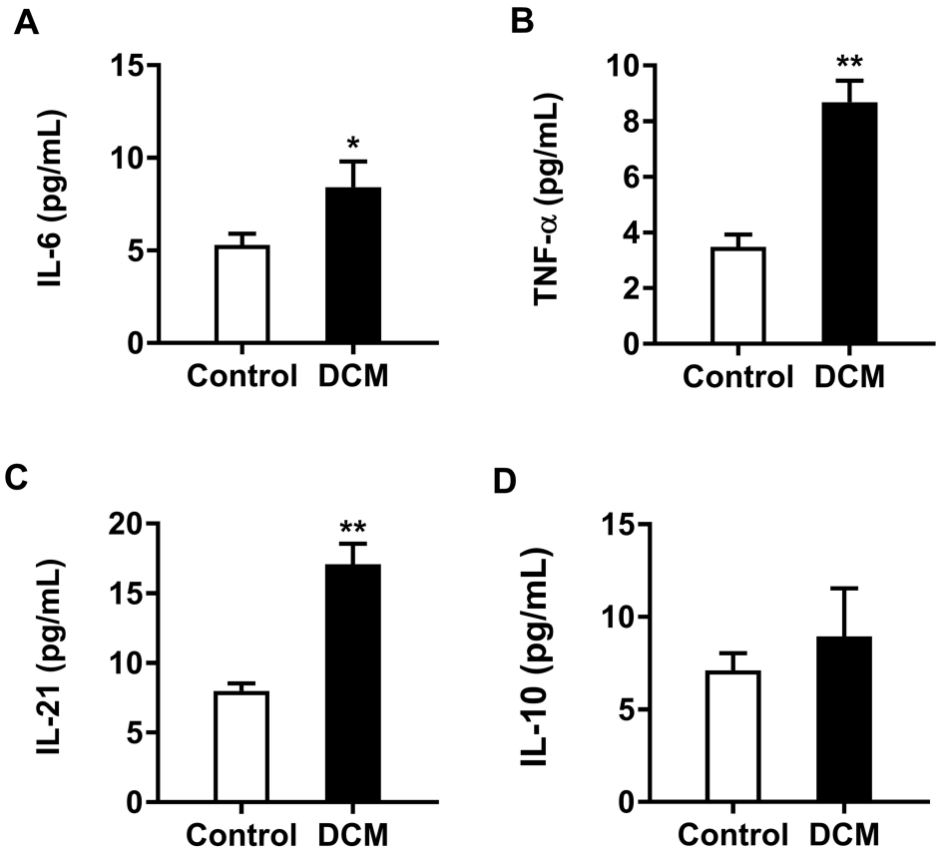
The levels of interleukin (IL)-6 (**A**, n=16), tumor necrosis factor (TNF)-α (**B**, n=30), IL-21 (**C**, n=30), and IL-10 (**D**, n=30) in plasma of patients with dilated cardiomyopathy (DCM) and healthy controls. Data are reported as means±SE. *P<0.05, **P<0.01 compared to the Control group (*t*-test).

ELISA detection showed that IL-6, IL-21, and TNF-α levels in plasma specimens from patients with DCM were significantly elevated compared with those of healthy control patients (Figure 4A-C). However, IL-10 levels were similar in both groups (Figure 4D).

### Associations of cytokines levels with Tfr and Tfh cells rates and Tfr/Tfh in DCM patients

**Figure 5:**
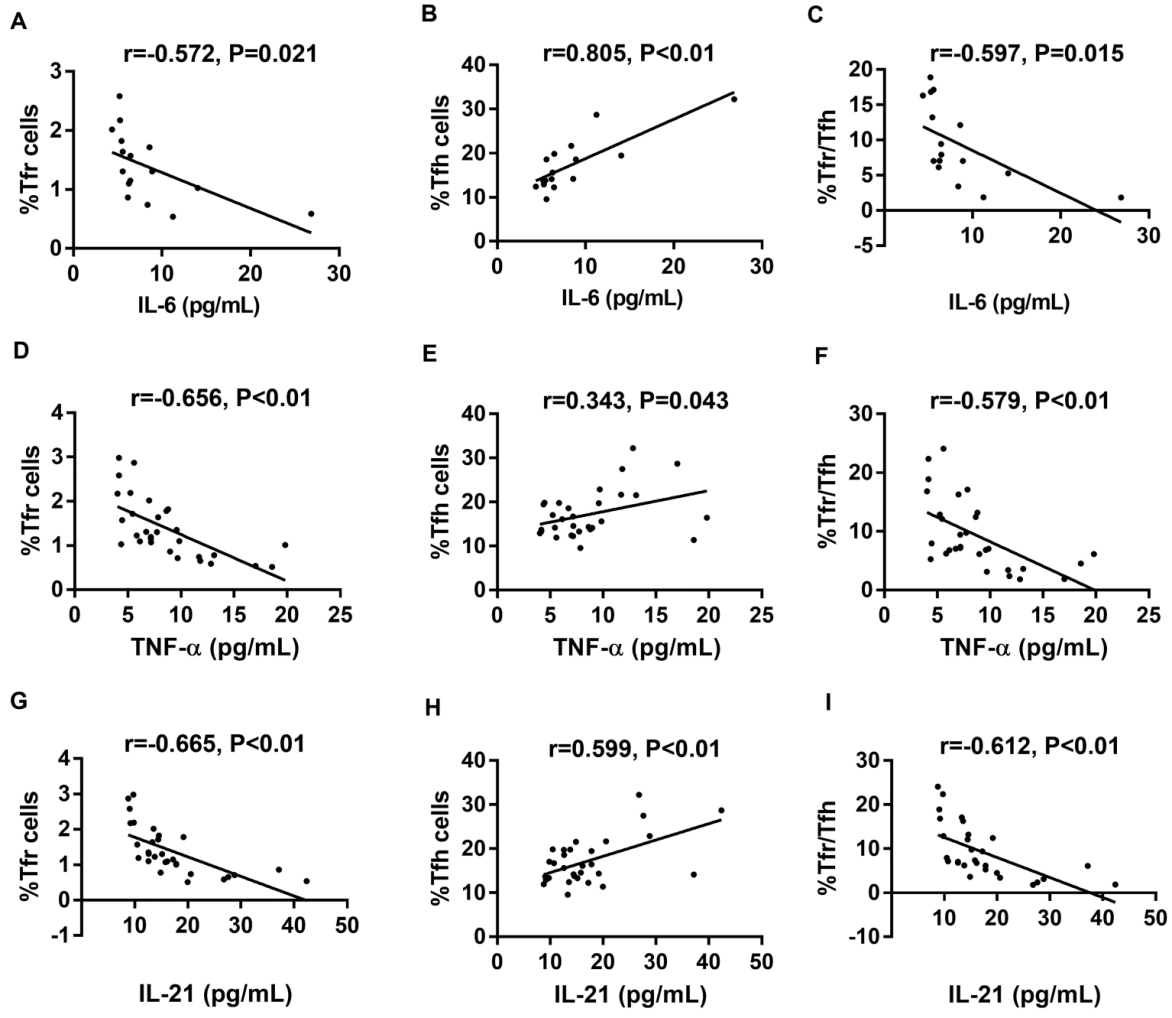
Correlation analysis between Tfr and Tfh cells rates and Tfr/Tfh with the level of cytokines in dilated cardiomyopathy (DCM) patients. Tfr cell rate was negatively correlated with interleukin (IL)-6 (**A**, n=16), tumor necrosis factor (TNF)-α (**D**, n=30), and IL-21 (**G**, n=30). Tfh cell rate was positively correlated with IL-6 (**B**, n=16), TNF-α (**E**, n=30), and IL-21 (**H**, n=30). Meanwhile, Tfr/Tfh was negatively correlated with IL-6 (**C**, n=16), TNF-α (**F**, n=30), and IL-21 (**I**, n=30).

As shown in Figure 5A-I, the Tfr cell rate was negatively correlated with IL-6, TNF-α, and IL-21. The Tfh cell rate was positively correlated with IL-6, TNF-α, and IL-21. Meanwhile, Tfr/Tfh was negatively correlated with IL-6, TNF-α,and IL-21.

### Increased levels of IgG and IgG3 in DCM patients

**Figure 6:**
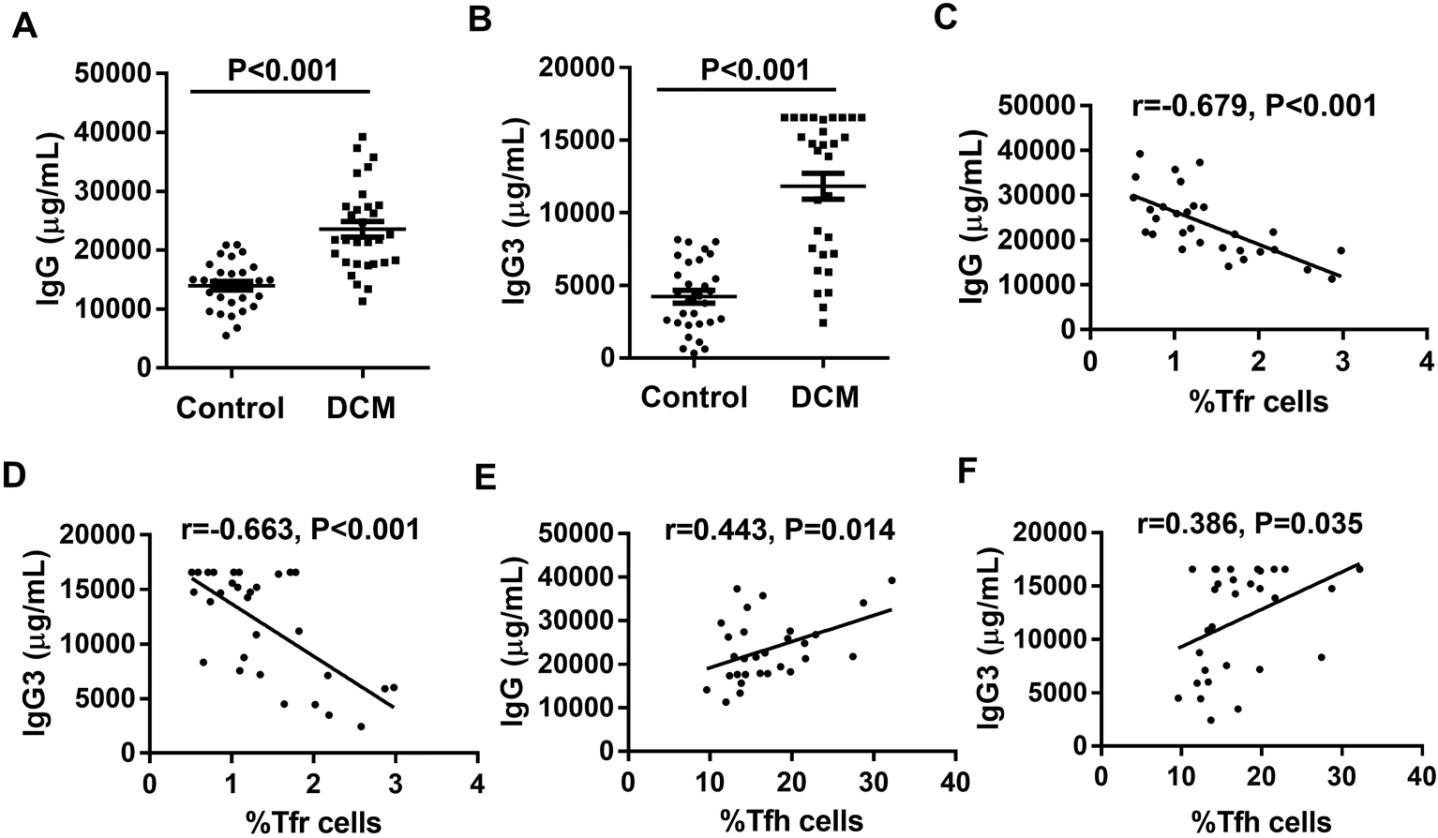
Plasma levels of IgG (**A**) and IgG3 (**B**) in healthy controls and dilated cardiomyopathy (DCM) patients. Pearson correlation analysis of Tfr frequency, Tfh frequency, and plasma IgG and IgG3 expression levels in DCM patients. The frequency of CD4^+^CD25^+^CXCR5^+^CD127^-/lo^ Tfr was negatively correlated with IgG (**C**) and IgG3 (**D**), while the frequency of CD4^+^CXCR5^+^CD127^+^ Tfh was positively correlated with IgG (**E**) and IgG3 (**F**).

We next compared plasma IgG and IgG3 levels between the two groups by ELISA. The results showed that IgG and IgG3 levels in plasma samples from DCM cases were significantly elevated compared with control levels (Figure 6A and B). These findings indicated that humoral immune response contributed to disease progression in patients with DCM.

### Associations of IgG and IgG3 levels with Tfr and Tfh cell rates in DCM patients

Finally, associations of Tfr and Tfh cell rates with IgG and IgG3 levels in DCM patients were assessed. As shown in Figure 6C and D, the Tfr cell rate was negatively correlated with IgG and IgG3. The CD4^+^CXCR5^+^CD127^+^ Tfh cell rate was positively correlated with IgG and IgG3 (Figure 6E and F).

## Discussion

Among the many pathogeneses of DCM, such as viral infection, immune damage, family inheritance, and oxygen free radical damage, immune impairment plays a vital role, especially the dysfunction of humoral immunity (17). CD4^+^CD25^+^CXCR5^+^CD127^-/lo^ Tfr cells constitute a newly discovered subgroup of Tregs expressing Foxp3. They can regulate germinal center response by controlling the levels of Tfh and B cells and play a critical role in maintaining immune tolerance and regulating immune activation (18,19). In DCM pathogenesis, active inflammation and persistent immune activation may reduce the levels of Tfr; then, Tfh and germinal center B cell proliferation and activation are out of control, as well as the production of related antibodies, which accelerates myocardial injury in patients with DCM (20). This study aimed to demonstrate the importance of changes in Tfr frequency and Tfr/Tfh ratio in DCM.

In recent years, the associations of Tfr rate changes with autoimmune diseases have been widely reported (11). In this study, it was found that the frequency of Tfh increased, the proportion of Tfr cells decreased, and the ratio of Tfr/Tfh decreased in patients with DCM. At the same time, increasing studies have confirmed that T cell activation and the production of myocardial autoantibodies are closely related to the deterioration of cardiac function of DCM (5,21). NT-proBNP, as a specific indicator for predicting adverse cardiovascular events, is positively correlated with LVEDD and left ventricular volume, and negatively correlated with LVEF (22). The level of its expression can reflect the severity and prognosis of heart disease. Heart failure and cardiac insufficiency caused by arrhythmia and cardiomyopathy can increase the expression level of NT-proBNP in patients' plasma, and the concentration of NT-proBNP increases with the severity of heart failure (23). At the same time, it was also confirmed in animal experiments that the level of NT-proBNP increased significantly within a few minutes after fatal arrhythmia (24). The same result also occurs in patients with atrial fibrillation (25). Therefore, in order to further evaluate the possible role of Tfr changes in DCM, we analyzed the associations of Tfr and Tfh cell frequencies with cardiac function indexes in DCM patients. We demonstrated that Tfr cell rate had positive and negative correlations with LVEF and NT-proBNP, respectively. Meanwhile, Tfh cell frequency had negative and positive correlations with LVEF and NT-proBNP, respectively. These findings indicated that Tfr frequency change is closely related to the deterioration of heart failure in DCM patients. Therefore, the decrease in the proportion of Tfr cells may be involved in impaired immune homeostasis *in vivo*, promoting the activation of T and germinal center B cells in DCM patients, with large amounts of secreted inflammatory cytokines and immunoglobulins. This may lead to the deterioration of cardiac function and myocardial injury.

Increasing evidence supports that abnormal Tfh cell proliferation, B cell activation, and anti-myocardial autoantibody production in the underlying pathophysiology of DCM are closely related to changes in Tfr cells. Ding et al. (26) reported that IL-21 downregulates CD25 by increasing BCL-6 expression in Tfr cells, thereby reducing Tfr cell affinity to IL-2, leading to a decrease in Tfr cell levels and finally enhancing germinal center response. In addition, as a biomarker of Tfh cell activation, ICOS positively regulates humoral response and IL-21 biosynthesis (27). Meanwhile, PD-1 promotes B cell survival and plasma cell formation via interaction with PD-L1 and PD-L2 on germinal center B cells, and thymic Treg differentiation into Tfr cells could also be selectively inhibited by PD-1 signaling (28). This finding suggested that Tfr cells may make the proliferation of Tfh and B cells uncontrollable through the decrease of their levels, and continue to activate the immune system, thereby increasing the production of anti-myocardial autoantibodies and subsequently leading to myocardial damage and impaired cardiac function.

This characteristic of Tfr cells, which can inhibit Tfh and B cell proliferation and differentiation and secrete antibodies, is likely controlled by multiple molecules such as Foxp3, PD-1, ICOS, and BCL-6. As shown in Figure 3, PD-1 and BCL-6 mRNA levels in PBMCs were significantly increased in DCM patients compared with healthy control individuals, which provided indispensable evidence for the participation of PD-1 and BCL-6 in the proliferation of Tfr cells. In addition, this study also measured the mRNA expression levels of the costimulatory molecule ICOS, which is essential for the interaction between Tfh and B cells. The results revealed increased ICOS levels in DCM patients compared with the normal control group. Multiple reports have demonstrated that the decrease of Treg specifically expressing Foxp3 may lead to reduced autoimmune tolerance and autoimmune cardiomyopathy (29). In a study by Zhu et al. (5), it was confirmed that impaired CD4^+^LAP^+^Treg function in circulation in patients with DCM leads to a defect in the function of inhibiting B cell proliferation and autoantibody production, which promotes the progression of DCM. Our findings corroborated these studies. In comparison with healthy controls, DCM cases had decreased Foxp3 mRNA levels in circulating PBMCs, suggesting that the levels of Tregs are decreased in DCM patients. This change also has an adverse effect on the maintenance of immune homeostasis in DCM and accelerates disease progression (3).

It has been determined that chronic inflammation participates in DCM pathogenesis (4). To further examine the profiles of inflammatory cytokines in plasma samples from patients with DCM, we assessed IL-6, TNF-α, and IL-21 levels in plasma. As demonstrated above, IL-6, TNF-α, and IL-21 levels in the DCM group were significantly higher than those of the control group, and Tfr, Tfh cell rate, and Tfr/Tfh were correlated with the level of cytokines. It was previously confirmed that IL-6 depends on Bcl-6 expression in Tfh cells regulated by STAT3, which induces T cell differentiation into Tfh cells and promotes Tfh to secrete IL-21 (30). Moreover, IL-21 not only induces the differentiation of B cells into plasma cells, but also promotes the proliferation of germinal center B cells and antibody production through CD40L/CD40 interaction, thus participating in autoimmune disorders (31,32). On the other hand, high levels of IL-21 also promote Tfh cells to prevent Tfr cell proliferation *in vivo* and restore the activation of B cells by inducing the expression of BCL-6 (33). Due to excessive B cell proliferation and activation in patients with DCM, TNF-α secretion does not only induce the expression of IL-6 gene and protein in various cell types, but it also produces anti-myocardial autoantibodies that indirectly participate in cardiomyocyte fibrosis and aggravate the disease process (34,35). We also detected the expression levels of IL-10 in plasma samples from both groups. As an important inhibitory factor of survival and proliferation in germinal center B cells, the inhibitory function of IL-10 may be consistent with that of Tfr cells, but the above results showed that the DCM and healthy control groups had similar values.

Studies have confirmed that altered B cell activation and autoantibody levels in circulation have critical functions in DCM occurrence and evolution (36,37). Staudt et al. (38) found that anti-myocardial autoantibodies belong to the IgG component; IgG3, which has a serious pro-inflammatory effect in the IgG subclass, plays a major role. Later on, researchers also confirmed through animal experiments that cardiac function indexes (such as LVEF) and hemodynamics in DCM patients are significantly improved after total IgG or IgG3 was eliminated by immunoadsorption (39). In the current work, plasma IgG and IgG3 levels were significantly higher in DCM patients compared with healthy controls, and the frequencies of Tfr cells in DCM cases had negative correlations with IgG and IgG3 levels. Moreover, Tfh cell rate had positive correlations with IgG and IgG3 levels, in agreement with previous studies (38).

The potential limitations of this study should be mentioned. Indeed, the increased secretion of heart-specific autoantibodies caused by excessive proliferation of B cells may promote progressive injury in cardiomyocytes. Unfortunately, this study did not elucidate the functional changes of Tfr cells during the progression of DCM disease, which may help develop new therapies for DCM.

To sum up, this study firstly revealed that reduced the frequencies of CD4^+^CXCR5^+^CD25^+^CD127^-/lo^ Tfr cells and imbalanced Tfr/Tfh ratio in the peripheral blood of DCM patients may play an important role in DCM by participating in immunomodulatory reactions (21). Therefore, further in-depth studies at the cellular level are warranted to determine the exact mechanism by which Tfr deficiency affects DCM pathogenesis, and to assess whether such cells could be used as potential therapeutic targets in DCM.

## Supplementary Material

Click to view [pdf].
